# Nutritional, Functional, and Technological Characterization of a Novel Gluten- and Lactose-Free Yogurt-Style Snack Produced With Selected Lactic Acid Bacteria and Leguminosae Flours

**DOI:** 10.3389/fmicb.2020.01664

**Published:** 2020-07-17

**Authors:** Erica Pontonio, Susanna Raho, Cinzia Dingeo, Domenico Centrone, Vito Emanuele Carofiglio, Carlo Giuseppe Rizzello

**Affiliations:** ^1^Department of Soil, Plant and Food Science, University of Bari Aldo Moro, Bari, Italy; ^2^Celery SRL, Bari, Italy

**Keywords:** yogurt-style snack, gluten-free, lactose-free, lactic acid bacteria, fermentation, high nutritional value

## Abstract

Aiming at meeting consumers’ requirements for healthy foods, dietary needs (vegetarianism, lactose- and gluten-free), as well as the nutrition recommendations of the Health Authorities in terms of protein, fibers and bioactive compounds, the present study proposes a novel yogurt-style snack made with plant-derived ingredients. The biotechnological protocol includes the fermentation of a thermal-treated blend of cereal and legume flours by the selected lactic acid bacteria (LAB) *Lactoplantibacillus plantarum* DSM33326 and *Levilactobacillus brevis* DSM33325. The yogurt-style snack was characterized by protein and fiber concentration of 3 and 4%, respectively, and a low-fat content. Compared to the unfermented control, the yogurt-style snack was characterized by a significant higher concentration of free amino acids and lower contents of the antinutritional factors, i.e., phytic acid, condensed tannins, saponins and raffinose (up to 90%) mainly due to the LAB metabolic activity. Hence, an *in-vitro* protein digestibility of 79% and improvements of all the nutritional indexes related to the quality of the protein fraction (e.g., GABA) were achieved at the end of fermentation. According to the Harvard Medical School recommendations, the novel snack can be potentially classified as low-glycemic index food (53%). Antioxidant properties of the fermented snack were also improved by means of increased the total phenol content and radical scavenging activity. High survival rate of the starter LAB and a commercial probiotic (added to the snack) was found through 30 days storage under refrigerated conditions. The biotechnological protocol to make the novel snack here proposed is suitable for the large-scale application in food industry, giving a platform product with a peculiar and appreciated sensory profile.

## Introduction

The concept of functional food perfectly meets the current consumer awareness regarding the correlation between diet and well-being; indeed, the healthiness is one of the main factors driving the consumer food choices (Granato et al., 2010). In this framework, research is investing in the development of novel foods which have a positive effect on the human organism through the modulation of a specific physiological function (Gobbetti et al., 2010).

Although yogurt and fermented milks are the functional foods mainly requested by consumers, there is a growing demand for beverages with a yogurt-like formulation, but not milk-based, defined as “yogurt-like drinks”. The vegetarianism, lactose intolerance and hypercholesterolemia are some of the reasons driving the increasing demand for milk-alternatives (Prado et al., 2008). Moreover, beverages turned out to be the most promising food category to better satisfy consumer demands, as they can be easily distributed, stored in refrigerated conditions and allow the easy incorporation of nutrients, bioactive compounds and probiotics (Corbo et al., 2014). To date, several beverages, mainly based on cereals, have been developed (Kandylis et al., 2016). Cereals are a good source of carbohydrates, proteins, vitamins, minerals and fibers (Blandino et al., 2003) and may determine excellent rheological properties (following a starch gelatinization process) when used as a basic ingredient yogurt-like products (Blandino et al., 2003; Coda et al., 2012).

Nevertheless, the increase of the global prevalence of celiac disease (*circa* 1–2% of the entire population) and the nutrition recommendations of the Health Authorities (e.g., the inclusion of high biological value protein, fibers and bioactive compounds in the diet) make the legumes promising candidates to produce novel functional foods and beverages (Maphosa and Jideani, 2017). Nevertheless, the choice of the optimal technological process to produce legumes-based foods needs to consider the presence of antinutritional factors (ANF) (phytic acid, condensed tannins, saponins, α-galactosides, etc.) which reduce the bio-availability of nutrients (mainly proteins and minerals) and negatively affect the sensory profile of the products (Curiel et al., 2015).

Several technologies have been proposed to produce cereal-based yoghurt-like beverages; nevertheless, fermentation appears to be the most widely used since, along with the technological properties, naturally enhances the nutritional and sensory profiles as well as prolongs the shelf life perspective (Hugenholtz, 2013). Overall, at industrial scale, the fermentation is carried out using selected starter cultures, which, once inoculated drive the process making it controllable and reproducible (Kandasamy et al., 2018).

Lactic acid bacteria (LAB) are often used for this purpose in various food matrices such as milk, meat, vegetables and cereals due to beneficial contributions of their metabolic traits on the product quality (Gobbetti et al., 2019a). The intense proteolytic (Savijoki et al., 2006) and specific enzyme activities (e.g., tannase, β-glucosidase, etc.) as well as the acidification (lactic and acetic acids) occurring during fermentation of cereal-derived and other vegetable matrices lead to several improvement of the food matrices. Indeed, increased contents of bioactive compounds (amino acids, phenols, peptides) (Filannino et al., 2018), lower concentrations of the ANF, enhanced nutritional features (*in-vitro* protein digestibility, starch hydrolysis and lowering glycemic index) (De Angelis et al., 2009; Peyer et al., 2016), improved sensorial profiles (Peyer et al., 2016) and extended shelf-life (antimicrobial activity) (Angelov et al., 2006) often characterized the fermented foods.

In this study, a biotechnological protocol to produce a novel gluten-free, lactose-free and vegan yogurt-style snack is described. A blend of rice, lentil and chickpea flours was used as raw matrix for the snack production, then subjected to mild physical treatments and fermentation by using the selected starters *Lactobacillus plantarum* DSM33326 and *Levilactobacillus brevis* DSM33325 (formerly known as *Lactobacillus plantarum* and *Lactoplantibacillus brevis*, respectively) (Zheng et al., 2020). The main nutritional, functional and sensory properties of the fermented snack have been characterized. Aiming at defining the role of the selected starters, the features of the novel yogurt-style snack were compared to those of the unfermented matrix. Starters survival, suitability to be used as a probiotic carrier and shelf-life of the novel snack in refrigerated conditions were also investigated.

## Materials and Methods

### Raw Materials and Microorganisms

Commercial rice (Bioalimenta S.r.l., Italy), chickpea (*Cicer arietinum* L.) (Molino Favero Srl, Padua) and lentil (*Lens culinaris*) (Molino Favero Srl) flours were used in this study. Protein (total nitrogen × 5.7), lipids, moisture, total dietary fiber and ash were determined according to Approved Methods 46-11A, 30-10.01, 44-15A, 32-05.01, and 08-01.01 of the American Association of Cereal Chemists (American Association of Cereal Chemists, 2010). Carbohydrates were calculated as the difference [100- (proteins + lipids + ash + total dietary fiber)]. Proteins, lipids, carbohydrates, total dietary fiber and ash were expressed as % of dry matter (d.m.).

*L. plantarum* DSM33326 and *Le. brevis* DSM33325 (Rizzello et al., 2020 patent) belonging to the culture collection of the Celery Srl (Polignano a Mare, Italy) and deposited in the DSMZ (Leibniz-Institut DSMZ-Deutsche Sammlung von Mikroorganismen und Zellkulturen GmbH, Braunschweig, Germany) culture collection were used as starter for fermentation. The potential probiotic strain *Lacticaseibacillus rhamnosus* SP1 (formerly known as *Lactobacillus rhamnosus*) (supplied by Sacco Srl, Cadorago, CO, Italy), one of the best studied and documented probiotic strains, with more than 300 clinical studies that have proven its efficacy and safety (Segura-Badilla et al., 2020), was added to the novel snack to assess the survival under refrigerated conditions. Strains were routinely propagated in De Man, Rogosa and Sharpe (MRS, Oxoid, Basingstoke, Hampshire, United Kingdom) at 30° C for 24 h. When used as starters for fermentation, LAB were cultivated until the late exponential phase of growth was reached (*circa* 16 h), harvested by centrifugation at 9,000 × *g* at 4°C for 10 min, washed twice in 50 mM phosphate buffer (4° C, pH 7.0) and resuspended in the tap water (final cell density of *circa* 7 log10 cfu/g) used for making the snack. When used, *La. rhamnosus* SP1 cells cultivated and washed as described above, were resuspended in the snack at the end of fermentation.

### Biotechnological Protocol to Make the Novel Yogurt-Style Snack

#### Processing

**FIGURE 1:**
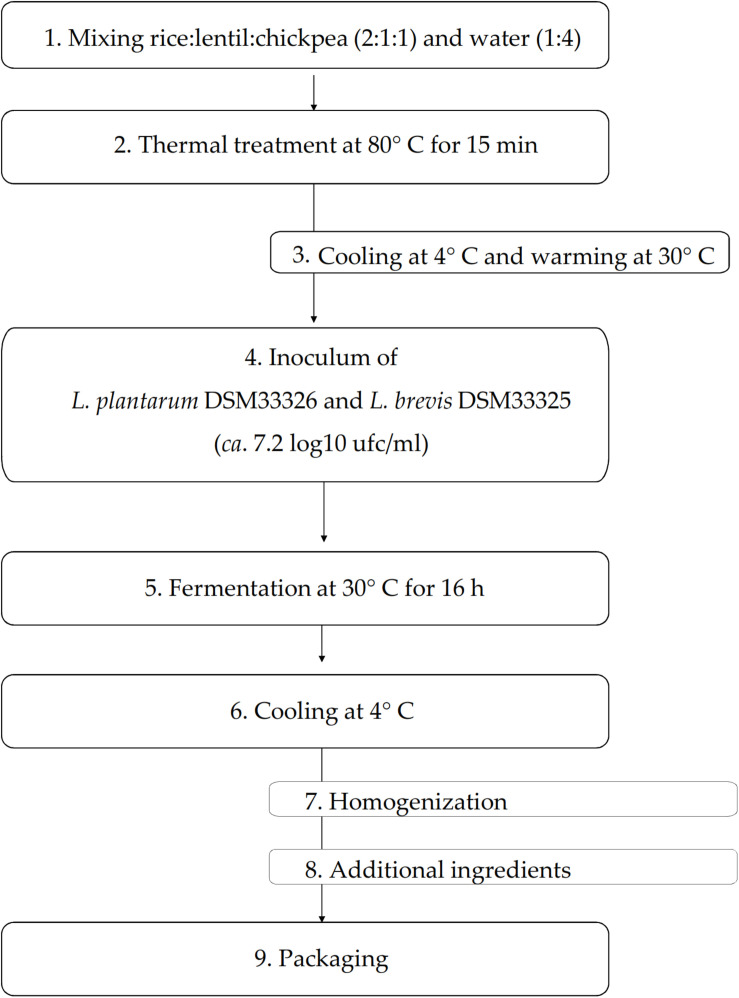
Flow-chart of the biotechnological protocol to make the novel yogurt-style snack.

The biotechnological protocol to make the yogurt-style snack (YS) is represented in Figure 1. Rice, lentil and chickpea flours were mixed (ratio 2:1:1) prior use. The mixture of flours was mixed (1:4) with tap water and homogenized with an Oster 6805 (Jarden Consumer Solutions Ltd., Cheadle, United Kingdom) mixer. Aiming at starch gelatinization, as described by Lindeboom et al. (2005), the mixture was treated at 80°C for *circa* 15 min under stirring conditions (70 rpm). Then, the mixture was first cooled down to *circa* 4° C in 2 min and then warmed up to 30° C prior the inoculum of *L. plantarum* DSM33326 and *Le. brevis* DSM33325 (ratio 1:1). The initial cell density of each strain was *circa* 7.2 log10 cfu/ml. The fermentation was carried out at 30° C for 16 h. At the end of fermentation, the mixture was cooled down to 4°C in 5 min, packaged in a glass jar and analyzed within 2 h after fermentation. A YS aliquot added with the potential probiotic *La. rhamnosus* SP1 (Coda et al., 2011) at the cell density of 9.7 log10 cfu/ml (pYS) was packaged and stored in the same conditions. The apparent viscosity of YS and cYS (yogurt-style snack prior the fermentation) was measured on about 35 ml of the snack, using the A & D SV-10 sine wave viscosimeter (A & D Company Ltd., Japan). Viscosity measurements were carried out on snacks previously maintained at 25° C for 30 min. Three independent productions were carried out and monitored. Samples were analyzed in triplicate.

#### Fermentation Monitoring

The pH of the snacks was determined by a M.507 pHmeter (Crison, Milan, Italy) equipped with a food penetration probe. Total titratable acidity (TTA) was determined on 10 g of snack homogenized with 90 ml of distilled water and expressed as the amount (ml) of 0.1 M NaOH to reach pH of 8.3. To determine the cell density of LAB, ten grams of sample were suspended in 90 ml of sterile sodium chloride (0.9%, wt/vol) solution and homogenized in a Bag Mixer 400P (Interscience, St Nom, France) at room temperature. Serial 10-fold dilutions were then plated into MRS (Oxoid) supplemented with cycloheximide (0.1 g/l) and incubated at 30° C for 48 h.

Kinetics of growth were determined and modeled in agreement with the Gompertz equation, as modified by Zwietering et al. (1990): y = k + A exp{- exp[(μmax or Vmax e/A) (λ-t) + 1]}; where y is the growth expressed as Log cfu/g/h at the time t; k is the initial level of the dependent variable to be modeled (Log cfu/g); A is the cell density (between inoculation and the stationary phase); μmax is the maximum growth rate expressed as ΔLog cfu/g/h; λ is the length of the lag phase measured in hours. Kinetics of acidification were modeled through a negative Gompertz equation. The fitting parameters were modified as follows: negative value of μmax was assumed as the maximal acidification rate (d max, ΔpH/h); λ, the lag phase, as the time (h) before the beginning of the decrease of pH value (α; metabolic adaptation time), and A as the maximum decrease of pH (ΔpH) (Bevilacqua et al., 2008). The experimental data were modeled by the non-linear regression procedure of the Statistica 12.0 software (Statsoft, Tulsa, United States).

### Characterization

#### Chemical and Nutritional Characterization

Major components and moisture of the snack were determined as reported for raw materials. Water/salt-soluble extract (WSE) from samples was prepared and used for the determination of organic acids, free amino acids (FAA) and γ-aminobutyric acid (GABA) concentrations. Organic acids in the WSE were determined by High Performance Liquid Chromatography (HPLC), using an ÄKTA Purifier system (GE Healthcare, Buckinghamshire, United Kingdom) equipped with an Aminex HPX-87H column (ion exclusion, Bio-Rad, Richmond, CA), and an UV detector operating at 210 nm. FAA and GABA were analyzed by a Biochrom 30 series Amino Acid Analyzer (Biochrom Ltd., Cambridge Science Park, United Kingdom) with a Na-cation-exchange column (20 by 0.46 cm internal diameter), as described by Rizzello et al. (2010).

#### *In-vitro* Protein Digestibility and Nutritional Indexes

The *in-vitro* protein digestibility (IVPD) was determined by the method of Akeson and Stahmann (1964), as reported by Rizzello et al. (2016). The IVPD was expressed as the percentage of the total protein, which was solubilized after enzyme hydrolysis.

Chemical Score (CS) estimates the amount of protein required to provide the minimal essential amino acids (EAA) pattern for adults, which was recently re-defined by FAO (Food and Agriculture Organization) in 2007 (Millward, 2012). It was calculated using the equation of Block and Mitchel (1946), which compares the content of EAA of the YSS for the same amino acid of the reference. The sequence of limiting essential amino acids corresponds to the list of EAA, having the lowest chemical score (Block and Mitchel, 1946). The protein score indicates the chemical score of the most limiting EAA that is present in the test protein (Block and Mitchel, 1946). Essential Amino Acids Index (EAAI) estimates the quality of the test protein, using its EAA content as the criterion. EAAI was calculated according to the procedure of Oser (1959). It considers the ratio between EAA of the test protein and EAA of the reference protein, according to the following equation:

$$\text{EAAI}=\sqrt[{\text{n}}]{\frac{\left({\text{EAA}}_{1}^{*}\,\,100\right)\,\left({\text{EAA}}_{2}^{*}\,\,100\right)\,\left(\ldots \right)\,\left({\text{EAA}}_{\text{n}}^{*}\,\,100\right)\,\left[\text{sample}\right]}{\left({\text{EAA}}_{1}^{*}\,\,100\right)\,\left({\text{EAA}}_{2}^{*}\,\,100\right)\,\left(\ldots \right)\,\left({\text{EAA}}_{\text{n}}^{*}\,\,100\right)\,\left[\text{reference}\right]}}$$

The Biological Value (BV) indicates the utilizable fraction of the test protein. BV was calculated using the equation of Oser (1959): BV = ([1,09 × EAAI] – 11,70). The Protein Efficiency Ratio (PER) estimates the protein nutritional quality based on the amino acid profile after hydrolysis. PER was determined using the model developed by Ihekoronye (1981): PER =−0,468 + (0,454 × [Leucine]) – (0,105^∗^[Tyrosine]). The Nutritional Index (NI) normalizes the qualitative and quantitative variations of the test protein compared to its nutritional status. NI was calculated using the equation of Crisan and Sands (1978), which considers all the factors with an equal importance: NI = (EAAI × Protein (%)/100).

#### Starch Hydrolysis and Predicted Glycemic Index

The analysis of starch hydrolysis (HI) was carried out on YS and cYS. The procedure mimicked the *in-vivo* digestion of starch (De Angelis et al., 2009). Aliquots of YS and cYS, containing 1 g of starch, were subjected to enzymatic process and the released glucose content was measured with D-Fructose/D-Glucose Assay Kit (Megazyme International Ireland Limited, Bray, Ireland). The degree of starch digestion was expressed as the percentage of potentially available starch hydrolyzed after 180 min. Wheat flour bread (WB) leavened with baker’s yeast was used as the control to estimate the hydrolysis index (HI = 100). The predicted GI was calculated using the equation: pGI = 0.549 × HI + 39.71 (Capriles and Areas, 2013).

#### Total Phenols and Antioxidant Activity

Total phenols were determined on the methanolic extract (ME) of samples prior and after the fermentation. Five grams of each sample were mixed with 50 ml of 80% methanol to get ME. The mixture was purged with nitrogen stream for 30 min, under stirring condition, and centrifuged at 4,600 × *g* for 20 min. MEs were transferred into test tubes, purged with nitrogen stream and stored at *circa* 4° C before analysis. The concentration was determined as described by Slinkard and Singleton (1977) and expressed as gallic acid equivalent. The radical DPPH% was used for determining the free radical scavenging activity (Rizzello et al., 2010). The synthetic antioxidant butylated hydroxytoluene (BHT) was included in the analysis as the reference (75 ppm).

#### Antinutritional Factors

Raffinose and phytic acid were determined using Megazyme kit K-PHYT 05/07 and Raffinose/D-Galactose Assay Kit K-RAFGA (Megazyme), respectively, following the manufacturer’s instructions. Total saponins were determined as reported by Lai et al. (2013) with minor modifications. Briefly, the freeze-dried samples (0.5g) were mixed with 10ml of petroleum ether by shaking for 4h. The residues (20 mg) were then extracted with 5 ml of 80% (vol/vol) aqueous methanol with shaking for 4h. The extracts were kept at 4° C in the dark until they were subjected to analysis. Total saponin content (TSC) was determined using the spectrophotometric method (Lai et al., 2013). All data are expressed on dry weight basis. Condensed tannins were determined using the acid butanol assay, as described by Hagerman (2002).

### Sensory Analysis

Sensory analysis of YS and cYS was carried out by 10 trained panelists (5 male and 5 females, mean age: 35 years, range: 18–54 years). After a roundtable discussion about the attributes, 16 attributes (Supplementary Table S1) were selected as the most frequently recognized by all the members of the panel. These were included in a score sheet for the quantitative evaluation with a scale from 0 to 10, with 10 the highest score. According to the IFST Guidelines for Ethical and Professional Practices for the Sensory Analysis of Foods, assessors gave informed consent to tests and could withdraw from the panel at any time, without penalty or having to give a reason.

### Shelf-Life, Starters and Probiotic Survival

Aiming at investigating the microbiological quality of the yogurt-style snack and the survival of the inoculated strains under refrigerated conditions, YS, pYS, and cYS were stored at 4°C for 30 days. The cell density of presumptive LAB, yeasts, molds and *Enterobacteriaceae* during storage (each 10 days) was determined as reported above. Yeasts were plated on Sabouraud Dextrose Agar (SDA, Oxoid), supplemented with chloramphenicol (0.1 g/l) at 25° C for 48 h. Molds were enumerated on Potato Dextrose Agar (PDA, Oxoid) at 25° C for 48 h. Total Enterobacteria were determined on Violet Red Bile Glucose Agar (VRBGA, Oxoid) at 37° C for 24 h.

To confirm the identity of the starter strains, LAB were isolated from YS, pYS and cYS after 10 (t1), 20 (t2) and 30 (t3) days of storage. At least 15 colonies of presumptive LAB were randomly selected from MRS plates containing the three highest sample dilutions. Gram-positive, catalase-negative, non-motile rods isolates were cultivated into the same media at 30° C for 24 h and re-streaked onto the same agar medium. All isolates considered for further analyses were able to acidify the culture medium. Genomic DNA from pure cultures of bacterial strains was extracted using a Bacterial Genomic DNA Isolation Kit 50 preps (Norgen Biotek Corp, Thorold, Canada), according to the manufacturer’s instructions. Three oligonucleotides, P4 5′-CCGCAGCGTT-3′, P7 5′-AGCAGCGTGG-3′, and M13 5′-GAGGGTGGCGGTTCT-3′, with arbitrarily chosen sequences, were used for bio-typing LAB isolates as reported by Nionelli et al. (2014b). RAPD-PCR (Randomly Amplified Polymorphic DNA-Polymerase Chain Reaction) profiles were acquired by the MCE-202 MultiNA microchip electrophoresis system (Shimadzu Italia S.R.L., Milan, Italy), using the DNA-2500 reagent kit (100 to 2500 bp) and the 2-logDNAladder (0.1 to 10.0 kb) (Promega S.R.L., Padua, Italy) according to the manufacturer’s instructions. The identity of *L. plantarum* DSM33326, *L. brevis* DSM33325, and *L. rhamnosus* SP1 were determined by comparing the RAPD-PCR profile of the inoculated strains with those of the LAB isolated during storage.

## Results

### Yogurt-Style Snack Production

The proximal chemical composition of flours used in this study is reported in Supplementary Table S2. Rice flour was characterized by a carbohydrates content *circa* 40% higher than the legumes. On the contrary, these latter contained circa 4-times higher protein than the rice. Chickpea was characterized by the highest content of dietary fiber (up to 4-times) and fat (up to 2-times) (Supplementary Table S2).

After 16 h of fermentation LAB reached a cell density of 9.8 ± 0.1 log10 cfu/g. LAB strains showed a lag phase of *circa* 2.4 h and μmax of 0.24 Δlog10 cfu/g/h. Along with the microbial growth, acidification was also monitored. Indeed, the pH values decreased from 6.50 ± 0.16 (cYS) to 4.04 ± 0.09 (YS). The decrease occurred with a Vmax of 0.3 dpH/h. TTA increased as results of the fermentation, indeed value of 4 ml NaOH 0.1 M higher than cYS were found in YS. Lactic and acetic acids had concentrations of 11.9 ± 0.2 and 5.4 ± 0.2 mmol/l, respectively, in YS, while they were not detectable in cYS (Table 1). The fermentation led to an increase of the apparent viscosity which was *circa* 14% higher than in cYS.

**TABLE 1:** Comparison of the main microbiological, biochemical and structural characteristics of the novel yogurt-style snack (YS) and the unfermented matrix (cYS).

	cYS	YS
***Microbiological properties***		
LAB (log10 cfu/ml)	2.3 ± 0.3^b^	9.8 ± 0.1^a^
***Biochemical characteristics***		
pH	6.50 ± 0.16^a^	4.04 ± 0.09^b^
TTA (ml NaOH 0.1M)	1.14 ± 0.20^b^	5.80 ± 0.15^a^
Lactic acid (mmol/l)	n.d	11.9 ± 0.2
Acetic acid (mmol/l)	n.d	5.4 ± 0.2
***Structural***		
Viscosity (Pa x s)	3.70 ± 0.13^b^	4.23 ± 0.11^a^

*Fermentation was carried out in triplicate at 30°C for 16 h. LAB, lactic acid bacteria; n.d., not detectable. The data are the means of three independent experiments ± standard deviations (*n* = 3). ^*a,b*^Values in the same column with different superscript letters differ significantly (*p* < 0.05).*

### Nutritional Characterization

The nutritional label of the novel yogurt-style snack is reported in Table 2. Overall, it was characterized by low fat (circa 0.57%) and high fiber (circa 4%) concentrations. Protein content was *circa* 3.2%.

**TABLE 2:** Proximal composition, nutritional indexes, IVPD, HI and pGI of the novel yogurt-style snack (YS) and the unfermented matrix (cYS).

	cYS	YS
***Proximal composition***		
Energy (Kcal)	68.0 ± 0.94	67.7 ± 1.05
Moisture (%)	79.27 ± 2.45	79.27 ± 2.45
Fat (g/100g)	0.58 ± 0.05	0.59 ± 0.09
Carbohydrates (g/100g)	12.80 ± 0.15	12.75 ± 0.12
Dietary fibers (g/100g)	4.12 ± 0.15	4.10 ± 0.20
Protein (g/100g)	3.27 ± 0.08	3.24 ± 0.16
Ash (g/100g)	0.40 ± 0.01	0.39 ± 0.02
***Nutritional indexes***		
Chemical Score		
Histidine	85 ± 1^b^	92 ± 1^a^
Isoleucine	65 ± 1	64 ± 1
Leucine	88 ± 1^b^	96 ± 2^a^
Lysine	113 ± 1	114 ± 1
Cysteine	42 ± 2^b^	55 ± 1^a^
Methionine	38 ± 1^b^	44 ± 2^a^
Phenylalanine + Tyrosine	49 ± 1^b^	63 ± 1^a^
Threonine	78 ± 1	78 ± 1
Valine	69 ± 1	70 ± 1
Tryptophan	44 ± 1^b^	62 ± 1^a^
Protein score	38 ± 1^b^	44 ± 2^a^
Biological Value (BV)	57 ± 2^b^	66 ± 2^a^
Protein Efficiency Ratio (PER)	33 ± 1^b^	36 ± 2^a^
Essential Amino Acid Index (EAAI)	63 ± 2^b^	71 ± 2^a^
Nutritional Index (NI)	1.70 ± 0.08^b^	2.77 ± 0.11^a^
TFAA (mg/l)	717.6 ± 14^b^	1181.9 ± 22^a^
IVPD (%)	67.3 ± 0.4^a^	79.5 ± 0.6^b^
HI (%)	38.2 ± 0.2^a^	25.1 ± 0.4^b^
pGI	60.6 ± 0.5^a^	53.4 ± 0.8^b^

*Fermentation was carried out in triplicate at 30° C for 16 h. TFAA, Total Free amino Acids, IVPD, in-vitro protein digestibility; HI, starch hydrolysis index; pGI, predicted glycemic index. The data are the means of three independent experiments ± standard deviations (*n* = 3). ^*a,b*^Values in the same row with different superscript letters differ significantly (*p* < 0.05).*

**FIGURE 2:**
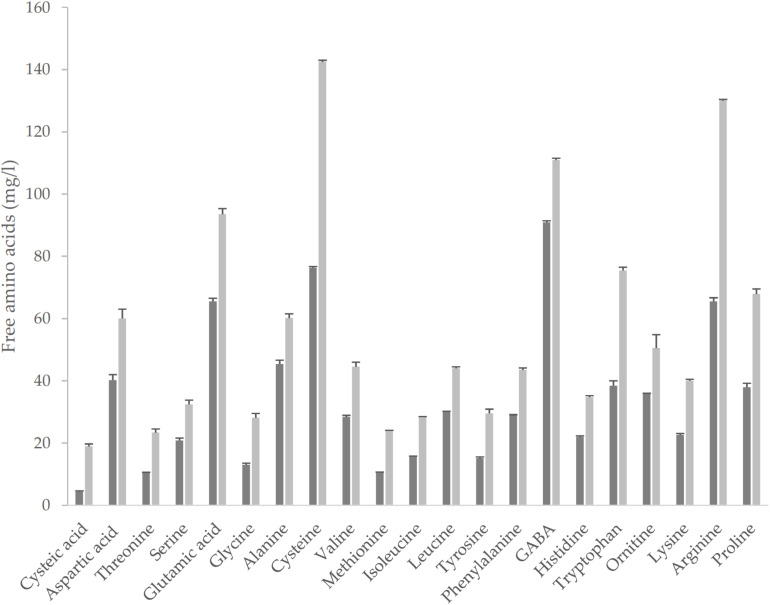
Free amino acids of the novel yogurt-style snack YS (light-gray bar) and of the unfermented matrix cYS (dark-gray bar). Fermentation was carried out in triplicate at 30° C for 16 h.

The fermentation led to an improvement of the nutritional profile of the matrix. Total FAA (TFAA) were *circa* 67% higher in YS as compared to cYS, as the consequence of the proteolysis operated by the selected starters on the native proteins. Significantly higher concentration of all the single amino acids were found in YS as compared to cYS (Figure 2), with a median increase value of *circa* 1.8-fold. The highest increases were observed for cysteic acid, Met, Thr and Gly concentrations (circa 2-fold compared to cYS), while the highest concentrations (>100 mg/l) were found for Cys and Arg (142.4 and 130.1 mg/l, respectively) (Figure 2).

The intense proteolysis well reflected on the IVPD. A multi-step protocol, which mimics the *in-vivo* gastrointestinal digestion, was used to estimate the snack protein digestibility, that resulted *circa* 18% higher for YS compared to unfermented cYS (Table 2). The digestible protein fraction was further characterized. In particular, the amino acid composition and the related CS were calculated, using the FAO reference. Based on CS, the sequence of limiting amino acids included Met, Cys and Trp, although the scores were significantly higher in YS compared to cYS. Indeed, the highest protein score was observed for YS (Table 2). EAAI and BV, which are commonly used to estimate the quality of food proteins, were higher for YS (circa 13 and 16%, respectively) compared to cYS (Table 2). The PER of YS was slightly higher than cYS. The NI, the only index calculated on the basis of the amount of digestible protein besides the amino acid composition, resulted *circa* 63% higher in YS than cYS (Table 2).

Values of HI, considered to be a presumptive measure of the GI in healthy subjects (Åkerberg et al., 1998), have been found to be *circa* 30% lower in YS than cYS. Accordingly, the pGI of YS was 53% (*circa* 13% lower than that of cYS) (Table 2).

The improvement of the nutritional profile was achieved also through the decreases of the ANF (Table 3). Indeed, the concentration of condensed tannins was *circa* 90% lower in YS as compared to cYS. Moreover, from 30 to 50% lower values were found for raffinose, phytic acid and saponins in YS compared to cYS (Table 3).

**TABLE 3:** Antinutritional compounds concentration of the novel yogurt-style snack (YS) and the unfermented matrix (cYS).

Anti-nutritional compounds (mg/100ml)	cYS	YS
Saponins	47.8 ± 0.7^a^	32.9 ± 0.6^b^
Condensed tannins	1.61 ± 0.05^a^	0.21 ± 0.01^b^
Phytic acid	69.2 ± 0.3^a^	35.5 ± 0.8^b^
Raffinose	18.2 ± 0.2^a^	10.7 ± 0.5^b^

*Fermentation was carried out in triplicate at 30°C for 16 h. n.d., not detectable. The data are the means of three independent experiments ± standard deviations (*n* = 3). ^*a,b*^Values in the same column with different superscript letters differ significantly (*p* < 0.05).*

### GABA and Antioxidant Activity

**FIGURE 3:**
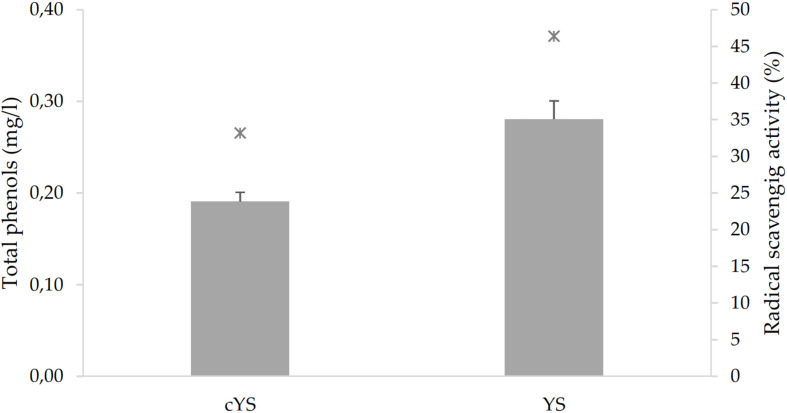
Total phenols (bars) and radical scavenging activity (stars) of the novel yogurt-style snack (YS) and the unfermented matrix (cYS). Fermentation was carried out in triplicate at 30°C for 16 h.

GABA, a functional non-proteic amino acid, was found in cYS at the concentration of 90 mg/ml. Fermentation led to a significant increase to the final content of 110.9 mg/l (Figure 2). Fermentation also caused the increase of the total phenol’s concentration (Figure 3). Overall, such compounds are the main responsible for the antioxidant activity of the food matrices. In particular, the concentration of total phenols in YS was *circa* 50% higher than cYS. Accordingly, the radical scavenging activity of YS was found to be *circa* 40% higher than that of cYS (Figure 3).

### Sensory Analysis

**FIGURE 4:**
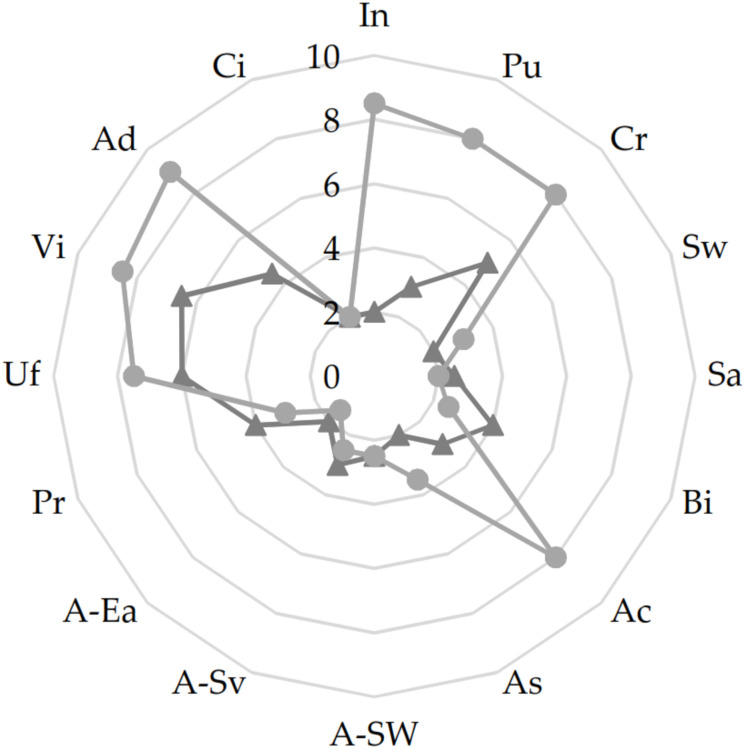
Sensory analysis of the of the novel yogurt-style snack YS (light-gray), and the unfermented matrix cYS (dark-gray). Fermentation was carried out in triplicate at 30°C for 16 h. In, intensity (odor); Pu, pungent (odor); Cr, creamy (odor); Sw, sweet (taste); Sa, salty (taste); Bi, bitter (taste); Ac, acidic (taste); As, astringent (taste); A-Sw, sweet (after taste); A-Sa, savory (after taste); A-Ea, earthy (after taste); Pr, particles (texture); Uf, uniformity (texture); Vi, viscosity (texture); Ad, adherence to spoon (texture); Ci, color intensity (visual).

The sensory profile of the cYS and YS was assessed. Overall, YS was characterized by sensory attributes (acidic and creamy odor and taste as well as the viscosity) considered optimal in yogurt products. Indeed, YS received high scores of pungent and creamy odors (mean value of 8/10) strongly affected by the presence of bacterial metabolites (fermentation) in the final products. Some of these, mainly organic acids, affected also the taste of YS which was found to be more acidic than the cYS (Figure 4). The viscosity and the degree of spoon adhesion were also enhanced through the biological acidification which affected the gel formation and maintenance. Indeed, higher values were found for YS as compared to cYS (Figure 4).

### Shelf Life and Probiotic Survival

The microbiological shelf-life of the yogurt-style snacks was assessed by monitoring the survival of molds, yeasts, Enterobacteria and LAB for 30 days storage (every 10 days) under refrigeration conditions. Supplementary Table S3 shows the trends on the microbial groups investigated. LAB in YS remained stable over time, with a cell density of *circa* 9 log10 cfu/l. The analysis of the RAPD-PCR confirmed the dominance of the two starters. No other LAB strains were present in YS at the end of fermentation and during storage at cell density higher than 4 log10 cfu/ml. An increase of the LAB density of *circa* 2.5 log10 cfu/l was found in cYS during storage. Increases of the cell density of yeasts and molds were also found in cYS, reaching value of 5.40 ± 0.67 and 5.06 ± 0.21 log10 cfu/l, respectively, after 30 days of storage. In YS, yeasts number reached value of 3.15 ± 0.23 log10 cfu/l while no growth was found for molds. Enterobacteria were found to be <10 cfu/10 ml in both cYS and YS (Supplementary Table S3).

Aiming at investigating the suitability of the YS to be used as carrier for probiotics, the survival of *La. rhamnosus* SP1 was also investigated. The cell density of the probiotic strain in pYS slightly decreased from *circa* 9 to 8 log10 ufc/l (Supplementary Table S3).

## Discussion

To date, the “healthy living” has been defined as the most impactful trend influencing the food industry (Gelski, 2019); indeed, consumers show preferences for minimally processed, closer-to-nature foods. Moreover, veganism and free-from foods trends are gaining popularity forcing industries to the production of novel foods (Costa and Johnson, 2019). Naturally nutrient-rich whole or minimally refined grains perfectly meet these needs and the industrial success in the commercialization of plant-based beverages suggest that novel uses of cereals, legumes and pseudocereals can provide a suitable alternative for consumer demands (Coda et al., 2017; Costa and Johnson, 2019).

Nevertheless, the protein in grains is not always comparable to traditional sources (mainly animal) and they often contain ANF which impair the nutritional properties as well as the palatability of these products. Fermentation, mainly with LAB, has been shown to be one of the most suitable unit operations used for processing grains since the onset of agriculture having a positive impact on the nutritional and sensory profiles of the grains-derived foods (Ganzle, 2019).

Here, a protocol to produce a milk-alternative yogurt-style snack made using gluten-free flours is described. The formulation, composed of rice, lentil and chickpea flours (2:1:1) and water (1:4) as well as the thermal protocol (80° C for 15 min) have been previously selected (Savijoki et al., 2006 patent) according to the optimal textural (e.g., viscosity) and sensorial properties of the products (data not shown). Moreover, the choice of the most suitable microbial starters, *L. plantarum* DSM33326 and *Le. brevis* DSM33325, was made according to their metabolic traits influencing the nutritional, chemical and sensory features of the final products (high acidification and proteolytic activity, capability to cause the degradation of ANF as observed in different legume-derived matrices) (Rizzello et al., 2020 patent).

The use of selected LAB in making novel plant-based beverages has already been recognized as the most efficient tool to drive and standardize the fermentation, to confer specific nutritional properties, such as the pre- and probiotic benefits, and to improve the sensory profile of the raw ingredients (Coda et al., 2017; Gobbetti et al., 2019a).

In this work, aiming at highlighting the effects of the fermentation, the nutritional, functional and technological features of a novel yogurt-style snack were compared to those of an unfermented matrix, however, obtained with the same formulation and technological processes.

Overall, the formulation allowed the obtainment of a low-calories and low-fat snack. Indeed, the energy value of the snack was 67.7 kcal on 100 g with circa 0.6% of fat. Rice flour was mainly used to confer a creamy structure, thanks to the abundance of gelatinizable starch. The use of lentil and chickpea flours allowed the enrichment in dietary fibers, that resulted markedly higher compared to the conventional milk-derived products, and the increase of protein concentration and quality compared to other cereal-derived beverages (Coda et al., 2017).

Dietary fibers were circa 4%, corresponding to 6 g on 100 kcal, while proteins were circa 3%, which covered more than 20% of the energy value of the product. According to European Regulation EC No. 1924/2006,2006, on nutrition and health claims on food products, the novel yogurt-style here proposed snack can be defined as low in fat, high in fiber and protein.

Along with the common characteristics associated to the lactic acid fermentation, i.e., decrease of pH due to the synthesis of lactic and acetic acids, the YS was characterized by enhanced nutritional and functional properties as compared to the mixture of the raw ingredients.

The dependence of the lactic acid bacteria on a complex proteolytic system to successfully ensure the fermentation processes, has widely been exploited for the improvement of the nutritional quality and bioavailability (peptides and free amino acids) of the protein in plant-derived matrices (Gobbetti et al., 2019a). In particular, the intense proteolysis operated by *L. plantarum* DSM33326 and *Le. brevis* DSM33325 during fermentation of the cereal/legume substrate led to a very high content of free amino acids (circa 1.1 g/l), that resulted more than 50-fold higher compared to a conventional milk yogurt (Tamime and Robinson, 1999), and 5-fold higher than a cereal yogurt-like beverage made with oat flakes (Nionelli et al., 2014a).

GABA, a functional amino acid present at relatively high concentration in legume flours (Nikmaram et al., 2017) and released from LAB through to the glutamate decarboxylase (GAD) activity (Renes et al., 2017), was at concentration higher than 100 mg/l in YS. Such concentration is potentially able to confer functional effects (Inoue et al., 2003). GABA is the major inhibitory neurotransmitter of the central nervous system and has several beneficial properties such as anti-hypertensive, prevention of diabetes, diuretic and tranquilizer effects (Diana et al., 2014).

As previously observed (Rizzello et al., 2019), proteolysis occurring during fermentation affected level of protein digestibility, that resulted, in the novel yogurt-style snack, circa 18% higher than the unfermented matrix. Aiming at investigating the protein quality of the novel yogurt-style snack, the digestible protein fraction was used for the determination of the protein quality indexes (Rizzello et al., 2014).

Thanks to the complementarity of the essential amino acid composition of the proteins from cereal (rice) and legume (lentil/chickpea) ingredients (Stone et al., 2019), YS had a very high EAAI. It resulted circa 54% higher than that found for a similar yogurt-like snacks made with quinoa, a pseudocereal overall known for the high quality of the protein fraction (Lorusso et al., 2018). Accordingly, all the indexes elaborated based on the comparison of the essential amino acid ratio to the FAO reference protein (Millward, 2012) resulted higher than those found for food products obtained from a single cereal, legume or pseudocereal matrix (Lorusso et al., 2018; Rizzello et al., 2019; Sozer et al., 2019). Within the indexes that are used to evaluate the nutritional value of foods, the NI combines qualitative and quantitative factors and is considered a global predictor of the protein quality. Since the higher protein bioavailability due to the extensive proteolysis caused by the selected starters during fermentation, the NI value of the yogurt-style snack here described was markedly higher than the unfermented control.

Besides the proteolysis caused by LAB, the higher value of the IVPD in the yogurt-style snack may also depend on the decrease of ANF, also occurring during fermentation. Indeed, legumes flours (lentil and chickpea), are overall characterized by the presence of ANF which may negatively affect the digestibility and the sensory properties of the derived products (Joye, 2019). Excluding trypsin inhibitors, that are thermolabile, condensed tannins and phytic acid are known to strongly complex the protein molecules, thus decreasing their bioavailability (Samtiya et al., 2020). Lactic acid bacteria fermentation has largely been proposed as tool to decrease such ANF; indeed, YS was characterized by 90% and 40% lower values of the condensed tannins and phytic acid, respectively, compared to the control. It was previously found that lactic acid bacteria can directly hydrolyze the complex tannins-protein through tannase activity (Pranoto et al., 2013) and can contribute to the phytic acid degradation providing microbial phytases (Karaman et al., 2018; Väkeväinen et al., 2018). Decreases of saponins and raffinose, due to the fermentation process have also been found. Although have some interesting biological properties (e.g., increase the permeability of the small intestinal mucosal cells, antifungal activity and lowering blood cholesterol) and industrial relevance (e.g., preparation of soaps, detergents, etc.), saponins are considered as antinutrient and flavor factors (Arendt and Zannini, 2013). Indeed, high concentration of saponins lead to decrease in mineral and vitamin bioavailability and astringent or bitter taste of the products (Cheeke, 2000; Tarade et al., 2006). YS contained circa 30% lower content of saponins compared to the control. Similar trend was found for raffinose, an α-galactoside not digested by pancreatic enzymes and metabolized by bacteria of the large intestine with gas production. Raffinose and similar galactosides present in legumes, like stachyose and verbascose, are subjected to enzymatic hydrolysis by LAB (Coda et al., 2017). Lower content (circa 50%) of raffinose in YS as compared to cYS may suggest an increased product digestibility and reduced digestive discomfort (Filannino et al., 2015).

Compared to the unfermented matrix, the concentration of total phenols in YS increased significantly. A similar phenomenon was already observed during legume fermentation by LAB (Gobbetti et al., 2019b), as the consequence of the combined effects of acidification, affecting solubility, and microbial hydrolytic enzymes that further promotes the enzymatic release of free phenolic compounds from glycosylated and more complex forms (e.g., trough feruloyl-esterase and β-glucosidase activities) (Filannino et al., 2015). As estimated toward DPPH radical, the increased concentration of total polyphenols observed after fermentation, corresponded to a proportional increase of the antioxidant activity.

The HI of YS was 30% lower than the unfermented matrix which led to a pGI of 53.4 ± 0.8. According to the Harvard Medical School recommendation which ranks foods with a GI ≤ 55 as low, between 56 and 69 as moderate and ≥ 70 as high GI foods, YS can be recognized as “low GI food”. Although promising value of the pGI were collected, in- vivo GI testing as described by the Regulation ISO 26642 (International Organization for Standardization) defined by FAO (Food and Agriculture Organization of the United Nations) and WHO (World Health Organization) need to be carried out. Overall, the gelatinization leads to changes in starch structure resulting in rapid degradation with release of fermentable sugars (Poutanen et al., 2009) from which mainly depend the HI and pGI of the cYS. LAB, fermenting sugars may have lowered the pGI of the product.

The quality of food products relies also on its the sensory acceptability as well as the shelf-life (Peri, 2006). Evaluation of the sensory profile and microbiological quality during storage have been carried out. Overall, YS was more like yogurt mainly due to the better odor, taste and texture characteristics.

Yogurt typically possess a smooth, viscous gel with a sharp acidic taste mainly due to the lactic acid (Das et al., 2019). The role of the fermentation was highlighted by the high scores (*circa* 8/10) of pungent and creamy odors which mainly rely on presence of organic acids, diacetyl and acetaldehyde, respectively, produced by microbial metabolisms (Wang et al., 2019). However, the role of proteolysis in the releasing of volatile compounds and precursors cannot be overlooked (Das et al., 2019). Organic acids, mainly lactic, release by the selected strains, also contributed to higher score of acidic taste in YS. Although it is not typically associated with the aroma profile of yogurt, lactic acid plays a vital role in the overall flavor profile of yogurt (Das et al., 2019). Moreover, the low pH (*circa* 4) of the YS might have contributed to the improved viscosity and adherence to spoon. Here, texture characteristics depend on the starch gelatinization and it was previously observed that pH values lower than 4 allow the preservation of the viscoelastic properties of the starch gel during long-storage period (Hirashima et al., 2012). The YS sensory profile was judged stable during the storage (data not shown), in agreement with previous studies showing the high sensory stability during shelf-life of vegetable beverages fermented with LAB (Peyer et al., 2016; Lorusso et al., 2018).

Moreover, the persistence of LAB at high cell density and the low presence of yeasts and molds in YS through the 30 days-storage under refrigerated condition suggest the stability of the microbiological quality. Although the determination of the main flours contaminant, *Bacillus cereus*, was not performed, the synergic effect of the refrigeration conditions and acidic environment is already reported as effective toward its spore germination and growth (Valero et al., 2003; Franceschini et al., 2020). Contrary to what observed in YS, a relevant presence of yeasts was found in cYS thus potentially compromising the quality of the product. However, under the study conditions, no undesirable odor and taste have been perceived by the panelists. The use of milk alternatives products as carrier of functional ingredients represents an interesting and promising approach to produce novel functional foods (Sethi et al., 2016). In this framework and aiming at evaluating the preliminary suitability of YS to be used as probiotic carrier, *La. rhamnosus* SP1, a well-known commercial probiotic strain, has been included in the formulation and its survival (during storage) investigated. The high cell density (*circa* 8 log10 ufc/l) found in the pYS at the end of the storage suggest that the YS may be considered adequate as vehicle for probiotics (WHO/FAO, 2002). However, further investigations on the role of the probiotic strains on the properties of the yogurt-style snacks may be required.

## Conclusion

Here, a novel gluten- and lactose-free yogurt-style snack was produced and characterized following an integrated approach including the evaluation of the main nutritional, functional, technological and sensory features. The novel product, conceived to be similar to conventional yogurt for the high density of viable LAB and the creamy structure (or texture), combines the advantages deriving by the use of a mixture of cereal and legume flours (high concentration of dietary fiber and an overall high biological value of the total proteins), and those deriving by the fermentation of the raw ingredients operated by selected strains of LAB (increase of the free amino acids and GABA content, increase of protein digestibility and nutritional indexes, enhancement of the antioxidant activity, decrease of the starch hydrolysis index and anti-nutritional compounds, and overall sensory appeal). The biotechnological protocol for making the novel snack here proposed is suitable for the large-scale application in food industry, giving a platform product largely customizable, for example with additional pro- and pre-biotics, and many other ingredients (e.g., fruit).

## Data Availability Statement

All datasets presented in this study are included in the article/Supplementary material.

## Author Contributions

EP carried out the data elaboration, writing, and review and editing of the manuscript. SR and CD carried out the formal analysis and data curation. DC and VC were responsible for the development of the biotechnological protocol for making the snack. CR conceived the experimental design and was the scientific advisor. All authors read and approved the final manuscript.

## Conflict of Interest

DC and VC are employed by Celery SRL. The remaining authors declare that the research was conducted in the absence of any commercial or financial relationships that could be construed as a potential conflict of interest.
