# Supplementary material for: Nutritional, Functional, and Technological Characterization of a Novel Gluten- and Lactose-Free Yogurt-Style Snack Produced With Selected Lactic Acid Bacteria and Leguminosae Flours
Source: Front Microbiol. 2020 Jul 17;11:1664. doi: 10.3389/fmicb.2020.01664 (PMC7379130; doi:10.3389/fmicb.2020.01664)
Supplement: Supplementary file 1 [file Data_Sheet_1.PDF]

Supplementary Table 1. List and definition of the attributes selected for the sensory analysis

Attributes	Abbreviation	Definition
Appearance and texture		
Color intensity	Ci	Intensity of the color (light to dark)
Uniformity	Uf	Uniformity of the texture at visual inspection
Adherence to spoon	Ad	Degree of spoon adhesion
Particles	Pr	Particles presence and bits (oral texture)
Odor		
Overall Intensity	In	Intensity of odor perceived immediately
Pungent	Pu	A sharp, even irritating physically penetrating sensation in the nasal cavity
Creamy	Cr	Odor associated to milk cream and butter
Taste		
Sweet	Sw	General taste factor associated with a sucrose solution
Salty	Sa	General taste associated to the presence of sodium salt
Bitter	Bi	Taste associated with caffeine and quinine
Acidic	Ac	Taste associated with lactic acid
Astringent	As	Sour, astringent, and slightly pungent aromatics associated with vinegar
After-taste		
Sweet	A-Sw	A lingering sweet syrupy flavor
Savory	A-Sv	Meaty, fleshy, beany flavor
Earthy	A-Ea	A lingering dirty, earthy, musty flavor

Supplementary Table 2. Proximal chemical composition of rice, chickpea and lentil flours

	Rice	Chickpea	Lentil
Chemical			
Energy (Kcal)	350	363	291

Fat (g/100g)	2.50±0.10^{b}	5.00±0.10^{a}	1.80±0.04^{c}
saturated (g/100g)	0.50±0.02^{b}	0.60±0.03^{a}	0.16±0.02^{c}
Carbohydrates (g/100g)	72.00±1.50^{a}	52.00±1.50^{c}	59.40±1.50^{b}
Dietary fibers (g/100g)	3.20±0.60^{c}	11.10±0.30^{a}	8.10±0.40^{b}
Protein (g/100g)	8.20±0.60^{c}	22.00±0.70^{b}	25.80±0.60^{a}
Ash (g/100g)	0.61±0.02^{c}	2.86±0.20^{b}	3.7±0.10^{a}

Data are expressed as % on dry matter. The data are the means of three independent experiments ± standard deviations (n=3). $^{a-c}$ Values in the same row with different superscript letters differ significantly (p<0.05).

Supplementary Table 3. Microbiological analysis of the unfermented matrix (cYS), novel yogurt-style snack (YS) and novel yogurt-style snack added with probiotic *Lactobacillus rhamnosus SP1* (pYS) carried out after production (0) and after 10, 20 and 30 days of storage at 4° C.

	**cYS**				**YS**				**pYS**			
Storage time (days)	0	10	20	30	0	10	20	30	0	10	20	30
LAB (log10 ufc/ml)	2.65±0.10	4.36±0.36	4.97±0.54	5.60±0.34	9.26±0.25	9.30±0.41	9.03±0.62	9.16±0.45	9.86±0.25	9.10±0.41	9.00±0.66	9.45±0.40
Enterobacteriaceae (log10 ufc/ml)	*	*	*	*	*	*	*	*	*	*	*	*
Molds (log10 ufc/ml)	*	*	3.11±0.34	5.06±0.21	*	*	*	*	*	*	*	*
Yeast (log10 ufc/ml)	2.67±0.26	3.26±0.14	4.24±0.54	5.40±0.67	*	*	*	3.15±0.23	*	*	*	*

LAB, lactic acid bacteria; The data are the means of three independent experiments ± standard deviations (n=3). $^{a-c}$ Values in the same row with different superscript letters differ significantly (p<0.05); * < 10 ufc/10 ml; n.i., not inoculated.
